# Virulence Profiles of Bacteremic Extended-Spectrum β-Lactamase-Producing *Escherichia coli*: Association with Epidemiological and Clinical Features

**DOI:** 10.1371/journal.pone.0044238

**Published:** 2012-09-07

**Authors:** Jesús Rodríguez-Baño, Jesús Mingorance, Natalia Fernández-Romero, Lara Serrano, Lorena López-Cerero, Alvaro Pascual, the ESBL-REIPI group

**Affiliations:** 1 Unidad Clínica de Enfermedades Infecciosas y Microbiología, Hospital Universitario Virgen Macarena, Sevilla, Spain; 2 Servicio de Microbiología, Hospital Universitario La Paz - IdiPAZ, Madrid, Spain; 3 Departamento de Medicina, Universidad de Sevilla, Sevilla, Spain; 4 Departamento de Microbiología, Universidad de Sevilla, Sevilla, Spain; Institut National de la Recherche Agronomique, France

## Abstract

There is scarce data about the importance of phylogroups and virulence factors (VF) in bloodstream infections (BSI) caused by extended-spectrum β-lactamase-producing *Escherichia coli* (ESBLEC). A prospective multicenter Spanish cohort including 191 cases of BSI due to ESBLEC was studied. Phylogroups and 25 VF genes were investigated by PCR. ESBLEC were classified into clusters according to their virulence profiles. The association of phylogropus, VF, and clusters with epidemiological features were studied using multivariate analysis. Overall, 57.6%, 26.7%, and 15.7% of isolates belonged to A/B1, D and B2 phylogroups, respectively. By multivariate analysis (adjusted OR [95% CI]), virulence cluster C2 was independently associated with urinary tract source (5.05 [0.96–25.48]); cluster C4 with sources other than urinary of biliary tract (2.89 [1.05–7.93]), and cluster C5 with BSI in non-predisposed patients (2.80 [0.99–7.93]). Isolates producing CTX-M-9 group ESBLs and from phylogroup D predominated among cluster C2 and C5, while CTX-M-1 group of ESBL and phylogroup B2 predominantes among C4 isolates. These results suggest that host factors and previous antimicrobial use were more important than phylogroup or specific VF in the occurrence of BSI due to ESBLEC. However, some associations between virulence clusters and some specific epidemiological features were found.

## Introduction

Most extraintestinal infections due to *Escherichia coli* are caused by isolates derived from the so-called virulent phylogenetic groups (PG) B2 and D, which exhibit more virulence factors (VF) than other PGs such as A and B1, hence considered “low virulence” or “commensal” PGs [1], [2]. Specifically, in studies on bloodstream infections (BSI) caused by *E. coli,* >70% of the isolates belonged to PGs B2 (which was predominant) and D [3]–[8]. Isolates from PG B2 have been associated with BSI with a urinary tract source [4], [5], [7] and in non-predisposed patients [7], [9], while PG A has been found with increased frequency in nosocomial BSI, compromised hosts [4], [6], [10] and in BSI caused by antibiotic-resistant isolates [5], [6], [8].

Some VFs have been assigned a pathogenic role in extraintestinal infections based on comparisons with rectal isolates, association with infections in non-predisposed patients, and animal models [11]. In studies dealing with BSI, several VF has been found to be associated with specific epidemiologic features, but it is *papGII* that has been more consistently associated with urinary tract sepsis as opposed to other sources, and in patients without predisposing factors [4], [9], [12].

Extended-spectrum beta-lactamase-producing *Escherichia coli* (ESBLEC) are increasing worldwide as a cause of community and nosocomial BSI, frequently affecting patients with predisposing conditions [12], [13]. There are scarce data about the distribution of PGs and VF in BSI due to ESBLEC [14] and their association with antimicrobial resistance. Also, to the best of our knowledge, the impact of specific VF in the epidemiology of BSI due to ESBLEC has not been studied. Finally, there is some controversy about the real virulence of ESBLEC, including isolates producing CTX-M-15 belonging to the worldwide spread clone ST131 [16]–[18].

The objectives of this study were to investigate the PGs and prevalence of VF genes in a well characterized collection of ESBLEC causing BSI, and whether some PGs and VF (individually or in clusters) were associated with the epidemiology, patients’ features and source of BSI.

## Methods

### Study Design and Patients

Data and isolates from a prospective cohort including 191 cases of BSI due to ESBLEC from 13 Spanish hospitals were used for this analysis. The epidemiology, clinical features, outcomes, types of ESBL and susceptibility data of this cohort were previously reported [13], [14]. Briefly, all monomicrobial BSI in patients with sign or symptoms of systemic infection caused by ESBLEC diagnosed in the participating hospitals between October 2004 and January 2006 were included. The cases were detected by daily review of microbiological results of blood cultures at each center. Data collected included demographics, acquisition classified as community, healthcare-associated or nosocomial [13], chronic underlying diseases, severity of underlying condition according to Charlson index [19], invasive procedures, exposure to antibiotics in the preceding 2 months, and source of BSI according to clinical and microbiological criteria.

**Table 1:** Virulence factor genes, ESBL groups and antimicrobial resistance of 191 ESBL-producing *E. coli* isolates causing BSI according to phylogroups.

	All isolates(n = 191)	Phylogroup B2(n = 30)	Phylogroup D(n = 51)	Phylogroups A/B1(n = 110)
Virulence score, median (IQR)	6 (4–8)	9 (8–13)^a^	7 (6–9)	6 (2–7)
VF				
*papC*	45 (24)	8 (27)	23 (45)^d^	14 (13)
*papGI*	0	0	0	0
*papGII*	31 (16)	3 (10)	20 (39)c,d	8 (7)
*papGIII*	7 (4)	4 (13)^a^	1 (2)	2 (2)
*sfaD/E*	4 (2)	1 (3)	1 (2)	2 (2)
*afaB/C*	13 (7)	9 (30)a,b	4 (8)^d^	0
*iha*	30 (16)	16 (53)a,b	10 (20)^d^	4 (4)
*fimH*	160 (84)	28 (93)^a^	48 (94)^d^	84 (76)
*hlyA*	4 (2)	3 (10)^a^	1 (2)	0
*cnf1*	4 (2)	3 (10)^a^	1 (2)	0
*cdtB*	4 (2)	3 (10)^a^	1 (2)	0
*sat*	39 (20)	19 (63)a,b	17 (33)^d^	2 (3)
*fyuA*	98 (51)	28 (93)a,b	37 (73)^d^	33 (30)
*iutA*	157 (82)	28 (93)^a^	46 (90)^d^	83 (76)
*iucD*	140 (73)	23 (77)	41 (80)	76 (69)
*iroN*	118 (62)	17 (57)	29 (57)	72 (66)
*kps MTII*	44 (23)	20 (67)a,b	19 (37)^d^	5 (5)
*traT*	141 (74)	28 (83)	37 (72)	79 (72)
*cvaC*	66 (35)	7 (23)	16 (31)	43 (39)
*ompT*	102 (53)	27 (90)a,b	32 (63)^d^	43 (39)
*ibeA*	15 (8)	7 (23)^a^	7 (14)^d^	1 (1)
*usp*	33 (17)	26 (87)a,b	7 (14)^d^	3 (3)
*maIX*	75 (39)	28 (93)a,b	34 (67)^d^	13 (12)
*svg*	3 (2)	2 (7)	0	1 (1)
*ireA*	29 (15)	3 (10)	19 (37)c,d	7 (6)
ESBL group^f^				
CTX-M-9 group^g^	122 (64)	10 (33)	41 (80)c,d	71 (65)
CTX-M-1 group^h^	42 (22)	17 (57)a,b	8 (16)	17 (16)
SHV group^i^	33 (17)	4 (13)	5 (10)	24 (22)
TEM group	1 (1)	0	0	1 (1)
Resistance to				
Cefotaxime	184 (96)	28 (93)	50 (98)	106 (96)
Ceftazidime	70 (37)	14 (47)^b^	12 (24)	44 (40)^e^
Cefepime	124 (65)	19 (63)	35 (69)	70 (64)
Amoxicillin/clavulanic acid	73 (38)	19 (63)a,b	20 (39)	34 (31)
Piperacillin/tazobactam	16 (8)	5 (17)	3 (6)	8 (7)
Ciprofloxacin	129 (68)	18 (60)	36 (71)	75 (68)
Gentamycin	39 (20)	4 (13)	14 (28)	21 (19)
Tobramicin	34 (18)	13 (43)a,b	10 (20)	11 (10)
Amikacin	3 (2)	2 (7)^a^	1 (2)	0
Co-trimoxazole	115 (60)	19 (63)	38 (75)^d^	58 (53)
Resistance score, median (IQR)	5 (4–7)	6 (4–7)^a^	5 (4–6)	5 (4–5)

a Higher in B2 vs A/B1 (p<0.05).

b Higher in B2 vs D (p<0.05).

c Higher in D vs B2 (p<0.05).

d Higher in D vs A/B1 (p<0.05).

e Higher in A/B1 vs D (p<0.05).

All other comparisons, p>0.05.

f 7 isolates produced >1 ESBL.

g Mainy CTX-M-14.

h Mainly CTX-M-15.

i Mainly SHV-12.

Data are presented as number of isolates (percentage) except where specified.

For this analysis, patients with any of the following were considered to have systemic predisposing features for BSI: diabetes mellitus, liver cirrhosis, chronic renal insufficiency, inmunosuppresive therapy, and neutropenia. Patients with a procedure-associated BSI (including vascular catheter, urinary catheter, endoscopic procedures and surgery), or any urinary or biliary tract BSI in the presence of obstructive diseases of these tracts were considered to have local predisposing factors for BSI. The study was approved by the Ethics Committee of Hospital Universitario Virgen Macarena which waived the need to obtained consent because all data were analysed anonymously and the observational nature of the study.

### Microbiological Studies

Methods for bacterial identification, susceptibility studies and ESBL confirmation and characterization were previously reported [13], [14]. Briefly, ESBL production and susceptibility by micro-dilution to cefuroxime, cefotaxime, ceftazidime, cefepime, amoxicillin-clavulanic acid, piperacillin-tazobactam, ciprofloxacin, gentamycin, tobramycin, amikacin, ertapenem, imipenem, meropenem, trimethoprim-sulfamethoxazol, fosfomycin, and tigecycline were studied according to CLSI recommendations [20]; a resistance score (number of antimicrobials to which the isolate was resistant) was calculated for each isolate. β-lactamase characterization was carried out by isoelectric focusing, PCR of the *bla* genes, and sequencing. ST131 clone was studied by O25b typing [17] and analysis for allele 3 of *pabB* [21]; the phylogenetic group was determined by multiplex PCR [22].

**Table 2:** Comparison of predisposing features according to phylogroup among 191 patients with bacteremia due to ESBL-producing *E. coli*.

	All isolates(n = 191)	Phylogroup B2(n = 30)	Phylgroup D(n = 51)	Phylogroups A/B1(n = 110)
Age in years, median (IQR)	71 (55–78)	72 (58–82)	71 (58–78)	69 (54–77)
Male gender	107 (56)	20 (66.7)	28 (54.9)	59 (53.6)
Acquisition				
Community	23 (12)	4 (13.3)	3 (5.9)	16 (14.5)
Healthcare-associated	72 (37.6)	12 (40)	24 (47.0)	36 (32.7)
Nosocomial	96 (50.2)	14 (46.7)	24 (47.9)	58 (52.7)
Nursing home resident	12 (6.2)	2 (6.7)	7 (13.7)	3 (2.7)
Charlson index, median (IQR)	2 (1–4)	2.5 (1–4)	2 (1–5)	2 (1–4)
Diabetes mellitus	52 (27.2)	9 (30)	12 (23.5)	31 (28.2)
Chronic pulmonary disease	34 (17.8)	4 (13.3)	9 (17.6)	21 (19.1)
Cancer	55 (28.7)	4 (13.3)a,b	15 (29.4)	36 (32.7)
Liver cirrhosis	18 (9.4)	5 (16.7)	4 (7.8)	9 (8.2)
Chronic renal insufficiency	28 (14.6)	3 (10)	5 (9.8)	20 (18.2)
Inmunosuppresive therapy	27 (14.1)	4 (13.3)	9 (17.6)	14 (12.7)
Obstructive urinary disease	43 (22.5)	7 (23.3)	7 (13.7)^c^	29 (26.4)
Biliary tract disease	18 (9.4)	2 (6.7)	3 (5.9)	13 (11.8)
Neutropenia	10 (5.2)	1 (3.3)	3 (5.9)	6 (5.5)
Urinary catheter	66 (34.5)	13 (43.3)	17 (33.3)	36 (32.7)
Central venous catheter	53 (27.7)	5 (16.7)	12 (23.5)	36 (32.7)
Mechanical ventilation	8 (4.1)	1 (3.3)	3 (5.9)	4 (3.6)
Previous surgery	44 (20.9)	6 (20)	14 (27.5)	24 (21.8)
Predisposing factor, local	122 (63.8)	19 (63.3)	30 (58.8)	73 (66.4)
Predisposing factor, systemic	100 (52.3)	16 (53.3)	24 (47.1)	60 (54.5)
Predisposing factors, systemic or local	158 (82.7)	24 (80)	41 (80.4)	93 (84.5)
Previous antibiotic use, any	107 (56)	18 (60)	28 (54.9)	61 (55.5)
Fluoroquinolones	49 (25.6)	7 (23.3)	12 (23.5)	30 (27.3)
Cephalospororins	53 (27.7)	9 (30)	15 (29.4)	29 (26.4)
Amoxicillin/clavulanic acid	20 (10.4)	3 (10)	3 (5.9)	14 (12.7)
Source				
Urinary tract	90 (47.1)	12 (40)	25 (49)	53 (48.2)
Biliary tract	24 (12.5)	3 (10)	5 (9.8)	16 (14.5)
Others^d^	77 (40.3)	15 (50)	21 (41.2)	41 (37.3)

a P value for B2 vs A/B1 = 0.03.

b P value for B2 vs D = 0.09.

c P value for D vs A/B1 = 0.07.

All other comparisons, P value ≥0.1.

d Other sources were: unkown, 25 patients; intraabdominal (non-biliar), 24; respiratory tract, 10; catheter-related, 9; miscellaneous, 8.

Data are expressed as number of cases (%) except where specified.

Twenty-five genes codifying for putative VF were studied, including adhesins (*papC, papGI, papGII, papGIII, fimH, sfaD/E, afaB/C, iha*); toxins (*cnf1, cdtB, sat, hlyA*); related to iron acquisition (*iucD, iroN, iutA, ireA,* and *fyuA*); protectins (*kps MTII, traT, cvaC,* and *ompT*); and miscellaneous (*ibeA, maIX, svg,* and *usp*). The presence/absence of VF genes was studied by PCR using previously described primers [2], [23]–[31]. Total DNA was purified from each strain with the UltraClean Microbial DNA purification kit (MO BIO Laboratories Inc., Carlsbad, CA). DNAs were distributed in 96-well master plates and PCRs were done in 50 µL mixtures containing 5 µl (20 ng) template DNA, 0.2 µM of each primer, 0.2 µM mix dNTPs and 1U DNA polymerase (Biotools S. L., Spain) in 1X buffer with MgCl_2_. PCR conditions were as follows: 5 min at 95°C, followed by 30 cycles of 30 s at 95°C, 30 s at annealing temperature of each primer pair, 1 min at 72°C, and a final 5 min incubation at 72°C. The PCR products were analyzed by electrophoresis in 96-well agarose gels (VG-FAST, Fisher Bioblock Scientific) stained with GelRed™ (Biotium Inc.). A virulence score (number of VF genes) was calculated for each isolate. The similarity of the isolates according to their VF genotypes was studied by constructing a dendogram using the binary patters (0, 1) of VF for each isolate; clusters of isolates were identified using the Dice similarity coefficient. After reviewing the data obtained, a 70% similarity threshold was used after reviewing the data obtained (a 60% threshold was not discriminative enough, since only one cluster included 72.2% isolates; and a 80% threshold found only 4 clusters with >5 cases including 32.4% of the isolates).

**Figure 1:**
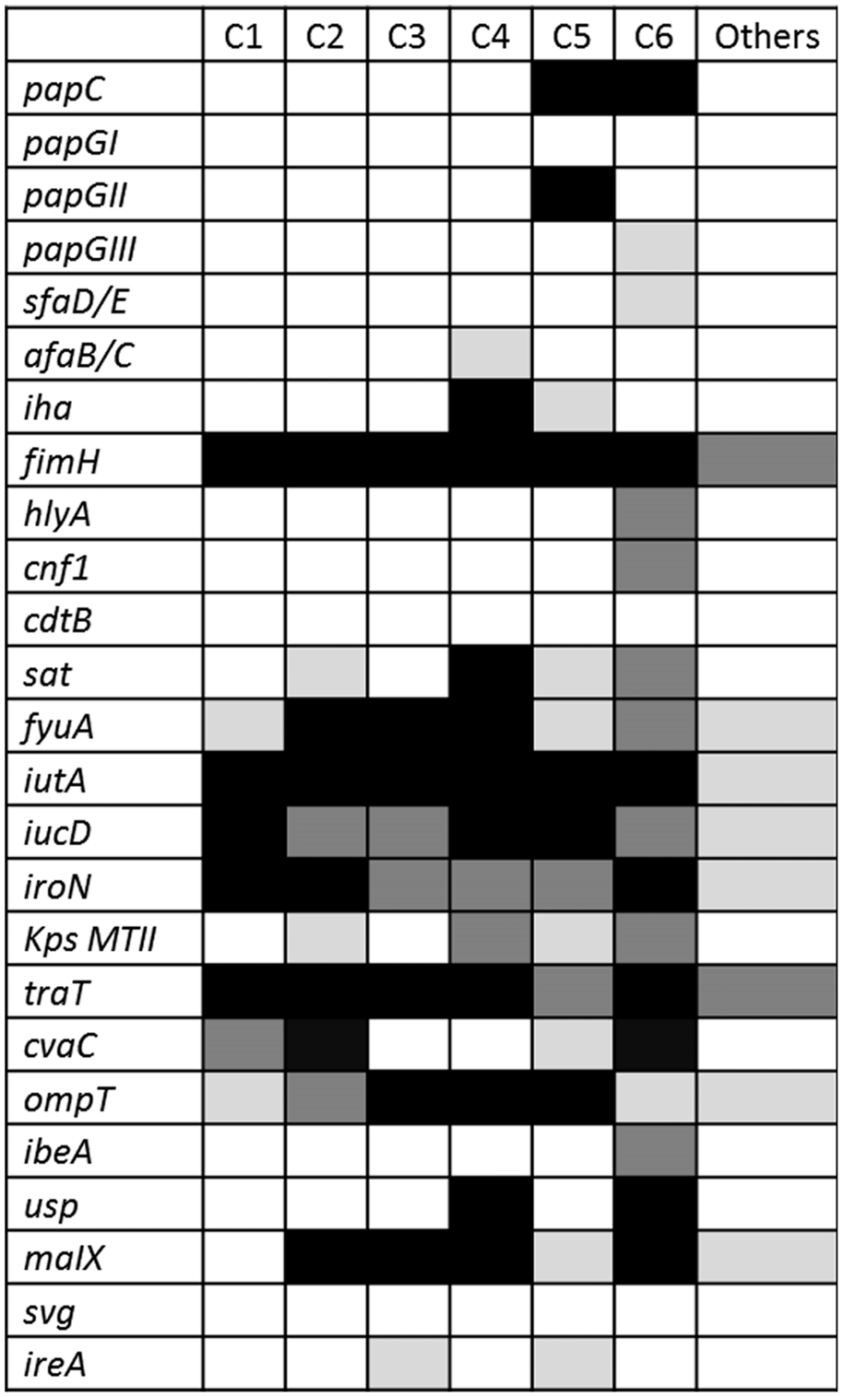
Distribution of virulence factors according to clusters. Percentage of isolates: white: 0–25%; pale grey, 26–50%; dark grey, 51–75%; black, >75%.

**Table 3:** Phylogroups, virulence score, ESBLs, selected antimicrobial resistance, and associated patients’ features of ESBL-producing *E. coli* isolates causing BSI according to virulence profile clusters.

	Cluster C1(n = 55)	Cluster C2(n = 11)	Cluster C3(n = 8)	Cluster C4(n = 22)	Cluster C5(n = 25)	Cluster C6(n = 7)	Other isolates (n = 63)
Phylogroups							
B2	0*	0*	25	90.9*	0*	57.1	6.3
D	10.9	54.4	75*	9.1	72*	42.9	15.9
A	23.6	18.2	0*	0*	16	0*	57.1
B1	65.5*	27.3	0*	0*	12	0*	20.6
Virulence score, median (IQR)	5 (5–6)*	8 (7–9)*	6 (6–7)	10 (10–12)*	8 (8–9)*	14 (12–15)*	4 (2–5)
ESBL groups							
CTX-M-1 group	16.4	18.2	50	68.2*	8	28.6	12.7
CTX-M-9 group	70.9	72.7	62.5	27.3*	84	57.1	61.9
SHV group	16.4	9.1	0	4.5	16	14.3	27
Antimicrobial resistance							
Ciprofloxacin	72.7	45.5	50	81.8	64	28.6*	69.8
Amoxicillin-clavulanic acid	29.1	45.5	37.5	68.2*	32	57.1	34.0
Tobramycin	7.3	18.2	12.5	54.5*	0*	14.3	22.2
Patients’ features							
Age in years, median (IQR)	68 (50–76)*	73 (66–78)	76 (70–83)	72 (57–79)	71 (52–77)	80 (58–84)	70 (58–80)
Male gender	58.2	54.5	50	63.6	48	42.9	57.1
Community-onset	50.9	45.5	62.5	50	56	28.6	47.6
Nursing home resident	1.8	9.1	12.5	13.6	12	0	4.8
Median Charlson index (IQR)	2 (2–5)	2 (1–5)	2 (1–4)	2 (2–4)	3 (2–4)	3 (1–3)	2 (1–4)
Predisposing factor, local	72.7	72.7	75	59.1	44*	85.7	60.3
Predisposing factor, systemic	60	37.3	50	50	40	71.4	54
Urinary tract source	52.7	81.8*	37.5	40.9	48	28.6	41.3
Biliary tract source	7.3	0	25	0	12	28.6	20.6
Other sources	40	18.2	37.5	59.1*	40	42.9	38.1

* P values <0.05 in comparison with isolates not included in the cluster.

Data are presented as percentage of isolates in each cluster except where specified.

### Statistical Analysis

Percentages were compared using the chi squared test or the Fisher exact test, as appropriate, and continuous variables using the Mann-Whitney U test. Multivariate analysis were performed by logistic regression; variables with a univariate p value <0.1 were introduced in the models, and selected using a stepwise backward process; 0.1 was set as the limit for removal of terms. All tests were performed using SPSS 18.0.

**Table 4:** Multivariate analysis of variables associated with specific sources of bloodstream infection.

	OR (95% CI)	P
Urinary tract source		
Age (per year)	1.02 (1.00–1.04)	0.009
Local predisposing factor	2.10 (1.11–3.98)	0.002
Systemic predisposing factor	0.57 (0.31–1.04)	0.07
Cluster C2	5.05 (0.96–26.48)	0.05
Non urinary or biliary tract sources		
Age (per year)	0.96 (0.94–0.98)	0.001
Local predisposing factor	0.27 (0.14–0.54)	<0.001
Systemic predisposing factor	2.75 (1.41–5.36)	0.003
Cluster C4	2.89 (1.05–7.93)	0.03

## Results

Among the 191 ESBLEC bacteremic isolates, 55 (28.8%) belong to PG A, 55 (28.8%) to PG B1, 51 (26.7%) belong to PG D, and 30 (15.7%) to B2. The median (IQR) virulence score were 10 (9–12) for B2, 8 (7–9) for D, 5 (4–6) for B1, and 4 (2–6) for A. Among the B2 isolates, 21 (70% of B2, 10.9% of the whole series) were O25b and *pabB*3 positive and were considered as belonging to ST131. For easier understanding, PGs A and B1 were analyzed together because both showed similar virulence scores, frequency of VF (only *fimH*, *iucD*, and *iroN* were significantly more frequent among B1 than among A), ESBLs (predominance of CTX-M-14), and resistance patterns.

**Table 5:** Multivariate analysis of variables associated with absence of local or systemic predisposing conditions.

	OR (95% CI)	P
Absence of systemic and local predisposing factors		
Age (per year)	0.97 (0.94–1.00)	0.04
Community-onset BSI	2.62 (1.02–6.76)	0.04
No previous antibiotics	5.69 (2.24–14.45)	<0.001
Cluster 5	2.80 (0.99–7.93)	0.05

The frequency of VF genes according to PG is shown in table 1. In summary, *papC, afaB/C, iha, fimH, sat, fyuA, iutA, kps MTII, ompT, ibeA, usp, and maIX* were more frequent among in B2 and D than in A/B1; additionally, *papGIII, hlyA, cnf1, and cdtB,* although infrequent, were more prevalent in B2 than in A/B1; and *papGII* and *ireA* were more frequent in D than in A/B1. Finally, *sfaD/E, afaB/C, sat, fyuA, kps MTII, ompT, usp*, and *maIX* were more frequent in B2 than in D, while only *papGII* was more frequent in D than in B2.

The distribution of the ESBL groups produced and resistance profiles to antimicrobials according to PG are also shown in table 1. ESBLs from the CTX-M-9 group (mainly CTX-M-14) were the most frequent among D and A/B1 isolates, while those from the CTX-M-1 group (mainly CTX-M-15) were the most frequent among B2 isolates. As regards antimicrobial resistance, isolates from the B2 PG showed a higher resistance score than A/B1 and were more frequently resistant to amoxicillin-clavulanic acid, tobramycin, and amikacin, while those from PG D were more frequently resistant to co-trimoxazole and less to ceftazidime.

Among the B2 isolates, 21 (70%) were ST131. The ESBLs produced by ST131 and non-ST131 B2 isolates differed; thus, CTX-M-15 was produced by 15/21 of ST131 isolates (71.4%) and by 0/9 of non-ST131 B2 isolates (p = 0.0007), while the numbers for CTX-M-14 were 2/21 of ST131 and 5/9 of non-ST131 B2 isolates (9.5% vs. 55.5%, p = 0.01). In comparison with non-ST131 B2 isolates, ST131 harboured more frequently *afaA/B* (42.9% vs. 0, p = 0.02), *iha* (66.7% vs. 22.2%, p = 0.04), and *sat* (81% vs. 22.2%, p = 0.004), and less frequently *papGII* (0 vs. 33.3, p = 0.02) and *ireA* (0 vs. 33.3%, p = 0.02).

The features of the patients according to PG are shown in table 2. Isolates from PG B2 and D did not seem to be related to lower frequency of predisposing features for invasive infections than isolates from PG A/B1. The only significant difference was cancer, which was less frequent among patients with B2 isolates than among those with A/B1. Also, there were not significant differences in the epidemiological features or sources of BSI. Even when B2 and D isolates were grouped, the only significant difference with A/B1 isolates was that the former more frequently occurred in nursing home residents (9/81 [11.1%] vs 3/110 [2.7%], p = 0.03).

The association of all 25 specific VF genes studied with predisposing factors for BSI, type of acquisition, previous antibiotic use, or source of BSI was studied. Overall, no association was found (data not shown) with 2 exceptions: *papGII* was more frequent in patients without any predisposing factor (local or systemic) than in patients with them (25% vs 12%, p = 0.02), while the opposite occurred with *sat* (25% vs 40%, p = 0.03). We also performed stratified analysis according to source. Among patients with a urinary tract source of BSI, those without any local or systemic predisposing feature had isolates with a higher prevalence of *papC* and *papGII* than those with any predisposing factor (46% vs 10%; p = 0.01, and 36% vs 7%; p = 0.001, respectively). No significant associations were found between VF and other sources of BSI.

The profiles of VF genes were extremely diverse: the 191 strains showed 159 different profiles, of these 134 were unique, 21 profiles appeared twice, 2 appeared three times, one profile was repeated four times and another one appeared five times. Such diversity prompted us to classify them in clusters; 29 clusters were found using a 70% similarity threshold; 6 clusters arbitrarily named C1–C6 grouped 128 isolates (67%). PGs, ESBLs, antimicrobial resistance, and epidemiological data according to cluster are shown in Table 3; distribution of VF among the clusters are shown in Figure 1. In summary, isolates from C1 caused infections in younger patients; those in C2 were associated with higher frequency of urinary tract source; C4 isolates showed higher frequency of CTX-M-1 group of ESBLs, resistance to amoxicillin/clavulanic acid and tobramycin, and bacteremia from sources other than urinary or biliary tracts; those in C5 had the lower frequency of local predisposing factors; and C6 isolates showed less frequent resistance for ciprofloxacin. Sixteen of the 22 isolates from C4 (73%) belonged to ST131; also, 76% isolates from ST131 belonged to C4.

To further investigate the association of C2 with urinary tract source, multivariate analysis were performed. We introduced the following variables: age, gender, acquisition, local predisposing factor, systemic predisposing factor, PG, cluster, *papGII*, VF score, ESBLs, and antimicrobial resistance score. C2 was independently associated with urinary tract source after controlling for age, local and systemic predisposing factors, while PGs, *papGII*, VF score or antimicrobial resistance were not (table 4). We did the same to investigate the association of C4 with sources other than urinary or biliary tracts. C4 was independently associated, while again PGs, specific VF, VF score, and antimicrobial resistance score were not (table 4).

Finally, we analyzed the association between different microbiological features and absence of predisposing systemic and local features for BSI. In the univariate analysis, cluster 5, *papC, papGII, sat,* female gender, lower age, community, source, and no receipt of previous antimicrobial use showed a p value <0.1 and were introduced in the multivariate analysis. The variables selected as independent predictors of BSI in non-predisposed patients were lower age, community-acquired BSI, no receipt of previous antimicrobials, and cluster 5 (table 5).

## Discussion

Our study showed that the phylogenetic background or virulence profiles of ESBLEC causing BSI in Spain were different to what would be expected for bacteremic *E. coli*. The fact that isolates from the so-called “low virulent” PGs A and B1 predominated as caused of BSI is in contrast with previous studies including mainly non-ESBL-producing isolates, in which B2 ad D were predominant [3]–[8]. As a consequence, the prevalence of all VF studied was much lower among ESBLEC isolates than among previous collections of blood isolates of *E. coli* except for *iutA, iroN, traT, and cvaC* [2]–[4], [6], [7], [12]. There are, to our knowledge, scarce previous data on collections of blood ESBLEC isolates. In a study from The Netherlands including 41 ESBLEC blood isolates, only 22% of isolates belong to A or B1 PGs [15]. Similar to our results, PG A was predominant in the subgroup of ESBL-producers from a French study; however, only 19 ESBLEC were included [8].

Three facts may explain our results. First, most cases occurred in patients with local or systemic predisposing factors for BSI; hence, less virulence factors would be required to cause invasive infection in such patients. Second, previous antibiotic treatment was common, which would have selected for ESBLEC because of their multidrug-resistant nature regardless their virulence profile. Although antimicrobial resistance has been frequently shown to be more frequent among isolates from the A and B1 PGs than among B2 isolates [11], B2 isolates were more frequently resistant to several antimicrobials (particularly amoxicillin-clavulanic acid and tobramycin) than isolates from other PGs. This reflects the resistance profile of isolates of ST131 producing CTX-M-15 [32], [33], which comprised most B2 isolates in our series. And third, in a recent study performed in France, non-ST131 B2 *E. coli* isolates were found to rarely produce CTX-M enzymes [34]; this, together with the fact that ST131 was not predominant in our series, would provide an additional explanation for the low rate of B2 isolates.

Even though ESBLEC from the B2 and D PGs showed, as expected, a much higher content in VF, we did not find B2 and D isolates to have caused infections in clearly less predisposed patients than A/B1 isolates, with the exception of cancer (less frequent among B2). A recent study on non-ESBL-producing *E. coli* found that B2 isolates were predominant as cause of bacteremia and spontaneous peritonitis in patients with liver cirrhosis [35]; of note, liver cirrhosis was more frequent among patient with B2 isolates than those with D or A/B1 isolates in our series, but the differences did not reach statistical significance. Also, we found that PGs or specific VFs were not independently associated to source of BSI. In previous studies of *E. coli* bacteremic isolates, those from PG B2 had been associated with urinary tract source of BSI [4], [5], [7]. As regards specific VF, several studies have investigated their association with BSI sources; the studies were different in populations, definitions, and VF studied, making it difficult to draw clear conclusions [4], [9], [12]. However, *papGII* has been more consistently associated with urinary tract source in these studies. We did not find such association, although *papGII* was more frequent in crude analysis among non-predisposed patients with urinary tract BSI.

Overall, these results suggest that host factors and previous antimicrobial use were more important than phylogroup background, virulence score or specific VF in the occurrence of BSI due to ESBLEC. However, by investigating the existence of clusters of isolates according to their VF content, we found some associations between virulence background and some specific epidemiological features. Thus, cluster C2 (mainly PG D, CTX-M-14 producers) was independently associated with urinary tract BSI; C4 (mostly B2 and ST131, CTX-M-15 producers) with non-urinary or biliary tract sources; and C5 (mostly D and CTX-M-14 producers) with BSI in non-predisposed patients. All these clusters had moderate to high virulence scores. The classification of isolates into clusters according to VF had been previously carried out by Johnson et al. according to clonal groups [36]; however, we constructed the clusters by considering exclusively the VF content of the isolates and without taking into account neither the phylogroups nor any other clonal relationship among isolates because our aim was to specifically investigate the influence of FV content by itself in the epidemiology of the infections. Hypothetical implications from our data are that vaccines developed against specific VFs might not be efficacious in avoiding invasive infections due to ESBLEC in predisposed patients, and that reducing the antibiotic pressure in such patients might be a more important measure to try and reduce such infections in these patients.

Strengths of our study include its multicenter nature, clinical data are comprehensive and were prospectively collected, and isolates are well characterized. However, it has some limitations: we could not compare the ESBLEC profiles with a control group of non-ESBL producers and thus used collections from other studies as a reference; we studied the genes codifying for VF, but this does not necessarily reflect the expression of these VF during infection; and results might not be applicable to areas with a different epidemiology of ESBLEC.

In conclusion, bacteremic ESBLEC more frequently belonged to PGs A and B1 and thus had a lower virulent content than expected; neither PGs or specific VF were consistently associated with predisposing features or sources of BSI; and some clusters of isolates identified according to their virulence profile were identified and associated with specific source or acquisition of BSI in the absence of predisposing factors.
